# Underwater Endoscopic Mucosal Resection of Small Rectal Neuroendocrine Tumors

**DOI:** 10.3389/fmed.2022.835013

**Published:** 2022-04-19

**Authors:** Masahiro Okada, Satoshi Shinozaki, Eriko Ikeda, Yoshikazu Hayashi, Takahito Takezawa, Hisashi Fukuda, Takaaki Morikawa, Masafumi Kitamura, Munefumi Arita, Tatsuma Nomura, Hirotsugu Sakamoto, Keijiro Sunada, Noriyoshi Fukushima, Alan Kawarai Lefor, Hironori Yamamoto

**Affiliations:** ^1^Division of Gastroenterology, Department of Medicine, Jichi Medical University, Shimotsuke, Japan; ^2^Shinozaki Medical Clinic, Utsunomiya, Japan; ^3^Department of Diagnostic Pathology, Jichi Medical University, Shimotsuke, Japan; ^4^Department of Surgery, Jichi Medical University, Shimotsuke, Japan

**Keywords:** endoscopic submucosal resection, neuroendocrine tumor, rectal neoplasms, patient outcome assessment (MeSH), underwater endoscopic mucosal resection

## Abstract

**Background and Study Aims:**

The resection strategy for rectal neuroendocrine tumors (NET) < 10 mm is not uniform. We compared the utility of underwater endoscopic mucosal resection (UEMR) to endoscopic submucosal resection with a ligation device (ESMR-L) to resect rectal NETs.

**Patients and Methods:**

Patients with rectal NET < 10 mm treated with UEMR or ESMR-L were included. Their medical records were retrospectively reviewed.

**Results:**

Thirty-two patients were divided into a UEMR group (*n* = 7) and an ESMR-L group (*n* = 25). Histopathological diagnosis of NET by biopsy was known before resection in 43% (3/7) in the UEMR group and 68% (17/25) in the ESMR-L group, (*p* = 0.379). UEMR was performed on an outpatient basis for all patients, and 92% of ESMR-L (23/25) were performed as inpatient procedures (*p* < 0.001). The procedure time was significantly shorter in the UEMR group than in the ESMR-L group [median (IQR), min, 6 (5–8) vs. 12 (9–14), *p* = 0.002]. *En bloc* resection and R0 resection rates were 100% in both groups. Pathological evaluations were predominantly NET G1 in both groups (UEMR: 7/7, 100% and ESMR-L: 23/25, 92%). Two patients in the ESMR-L group developed delayed bleeding, controlled by endoscopic hemostasis. Device costs were significantly higher in the ESMR-L group than the UEMR group by approximately US$180 [median (IQR), $90.45 (83.64–108.41) vs. $274.73 (265.86–292.45), *P* < 0.001].

**Conclusion:**

UEMR results in similar resection quality with shorter procedure time and lower costs compared to ESMR-L. We recommend UEMR for the resection of rectal NET < 10 mm.

## Introduction

**FIGURE 1:**
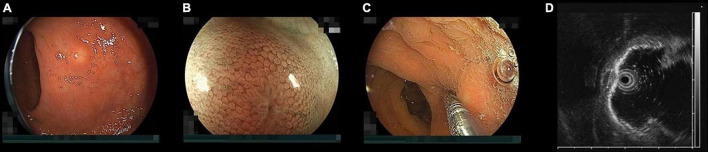
Endoscopic findings of a rectal neuroendocrine tumor (NET). **(A)** Typical rectal NET endoscopically looks like a yellow submucosal tumor covered with normal mucosa **(B)** magnified view shows “expanded normal pit pattern” because NETs originate from the deep mucosa and expansively grow toward the submucosa through the muscularis mucosa expanding the pits on the surface of the mucosa covering the NET. **(C)** When pushing a NET using the tip of a miniature probe, the NET moves with its mucosa but a submucosal tumor from the muscularis separately moves under the mucosa. **(D)** Endoscopic ultrasound at 20 MHz can correctly distinguish a NET from a submucosal tumor. The high-echoic submucosal layer can be identified between the NET and the muscularis. The NET was measured 5.2 mm in diameter with measuring function prepared to an endoscopic ultrasound unit.

Neuroendocrine tumors (NET) were formerly referred to as carcinoid tumors which means “cancer like” because NETs generally have slower progression and a lower rate of metastases than ordinary cancers (1). However, it is not rare that NETs develop distant metastases clinically. Therefore, the World Health Organization renamed them, especially in the gastrointestinal organs, to NETs in 2000 because of concern that the name carcinoid might lead to underestimating their biological potential (2). Typical rectal NETs appear endoscopically like submucosal tumors. However, small rectal NETs can be resected endoscopically when they are limited to the submucosa because NETs originate from the deep mucosa (Figures 1A–D). A rectal NET smaller than 1 cm and limited to the submucosa is an indication for endoscopic excision according to the Clinical Practice Guideline for Gastroenteropancreatic Neuroendocrine Neoplasms 2019 in Japan (3). Most rectal NETs are rarely limited to the mucosa because they grow into the submucosa breaking through the muscularis mucosa. Therefore, conventional hot snare polypectomy or endoscopic mucosal resection (EMR) rarely results in excision of a NET with a negative vertical margin. Although endoscopic submucosal dissection (ESD) can lead to excision of rectal NETs with a negative margin under the clear visualization of the submucosal layer (4), ESD has a higher cost and longer procedure time, and the Japanese health insurance does not cover ESD for rectal NETs smaller than 5 mm but does cover EMR. Rectal NETs smaller than 1 cm can also be removed using endoscopic submucosal resection with a ligation device (ESMR-L). Practically, ESMR-L is commonly used as an endoscopic-mucosal-resection technique when resecting small rectal NETs in Japan (5). However, dedicated ligation devices for ESMR-L are not available yet. ESMR-L is generally performed using a rubber band from a band-ligation kit consisting of multiple rubber bands dedicated to endoscopic variceal ligation in addition to EMR devices. After using only one band for an ESMR-L procedure, the several remaining bands are usually wasted. However, ESMR-L is still commonly chosen for removal of rectal NETs, especially those smaller than 5 mm, in practice in Japan because the total cost of ESMR-L is much lower than ESD.

Underwater EMR without submucosal injection (UEMR) was reported as a “game-changing” EMR technique for the resection of large sessile colon tumors by Binmoeller et al. (6). When exchanging gas for water in the colorectum, the colorectal mucosa and submucosa appear to shrink and float above the circular muscularis propria under endoscopic ultrasound view. Therefore, even a flat tumor can easily be captured by a loop snare including sufficient submucosa under it without submucosal injection. Yamashina et al. (7) reported the efficacy of UEMR for excision of rectal NETs. Park et al. (8) reported that the R0 resection rate using UEMR was as good as ESD and the procedure time for UEMR was shorter than ESD when resecting rectal NETs. If UEMR is as effective as ESMR-L for removal of rectal NETs, UEMR may remove rectal NETs more efficiently. The aim of this study was to assess the effectiveness and efficiency of UEMR compared with ESMR-L for resecting rectal NETs.

## Patients and Methods

A total of 74 NETs in 72 patients were endoscopically resected and pathologically diagnosed at Jichi Medical University Hospital between April 2015 and April 2021. Forty patients with NETs resected by ESD were excluded from this analysis. In patients with more than two NETs, only the first resected NET is included in this study. Finally, 32 patients with NETs were retrospectively analyzed in this study. Seven patients underwent UEMR and 25 patients underwent ESMR-L. Written informed consent for endoscopic resection of the NETs was obtained from all patients. This retrospective analysis was approved by the Institutional Review Board of Jichi Medical University (No. 20-103).

### Endoscopic System and Devices

When performing total colonoscopy and endoscopic ultrasound before resection, a magnification endoscope with a waterjet channel (EC-L600ZP or EC-760ZP-V/M; Fujifilm, Tokyo, Japan), a carbon dioxide insufflator (GW-1 or GW-100; Fujifilm), and a black cap (MAJ-1991; Olympus, Tokyo Japan) or a transparent distal attachment (D-201-14304; Olympus), a water irrigation system (JW-2; Fujifilm) with distilled water, diathermy (ESG-100; Olympus) and a 20 MHz miniature probe with an endoscopic ultrasound processor (UM-3R and EU-ME1; Olympus) were used.

### Endoscopic Examination

Patients underwent bowel preparation using 10 mL of oral 0.75% sodium picosulfate the night before colonoscopy and 2 L of polyethylene glycol electrolyte solution on the day of colonoscopy. When the stool became watery and clear, bowel preparation was considered complete even if the entire 2 L of polyethylene glycol solution had not been ingested. Midazolam and pethidine were used as sedation, and timepidium bromide hydrate or glucagon was used to decrease colonic peristalsis.

Even if NETs were found early in the colonoscopy, total colonoscopy was performed in all patients before examination of the NETs. Endoscopic ultrasound was also performed to characterize the NETs and to measure their size during colonoscopy.

We performed ESMR-L for small rectal NETs as inpatient procedures until the COVID-19 pandemic. However, we performed UEMR on an outpatient basis during the pandemic when admissions were restricted.

### Method of Underwater Endoscopic Mucosal Resection

**FIGURE 2:**
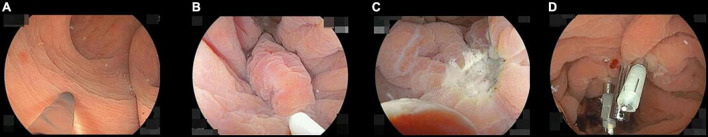
Pictures of the underwater endoscopic mucosal resection sequence. **(A)** Filling up the rectum with distilled water while completely aspirating residual gas. **(B)** Snaring the neuroendocrine tumor (NET) involving the surrounding mucosa and the submucosa under the NET. If they cannot be appropriately snared, snaring should be repeated until it is achieved. **(C)** When the specimen was resected, we confirm there are neither any residual tumor nor perforations under water immersion. **(D)** The wound was closed with endoclips during water immersion.

All UEMRs were performed under water immersion just after performing the endoscopic ultrasound without changing the colonoscope. If gas remained above the water, the gas was completely aspirated (Figure 2A). The tip of a 15 mm rotatable snare (Supplementary Video 1) (RotaSnare; Medi-Globe, Rohrdorf, Germany) was anchored to the proximal side of the NET to keep the NET at the center of the snare. When snaring the NET, water was aspirated to capture as much tissue around and under the NET as possible. If the NET was not snared with sufficient surrounding tissue, it was released and snared repeatedly until completely snared (Figure 2B). Subsequently, NETs were cut with 15 watts pure-cut mode diathermy (ESG-100; Olympus) (Figure 2C). The mucosal defect was closed using a reopenable endoclip (SureClip 16 mm; Micro-Tech, Nanjing, China) using underwater immersion. If the defect could not be closed completely or immediate bleeding could not be stopped, other endoclips (EZ Clip; Olympus) were added (Figure 2D).

### Method of Endoscopic Submucosal Resection With a Ligation Device

**FIGURE 3:**
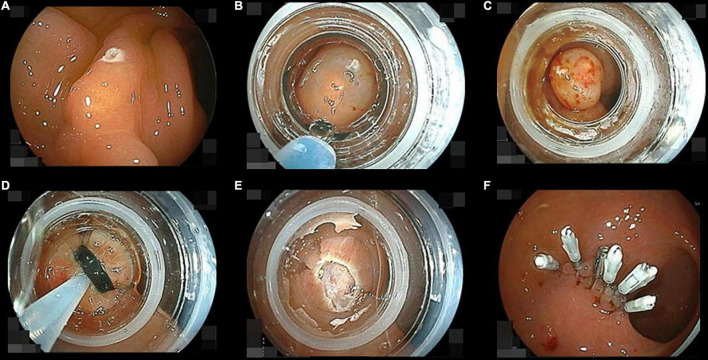
Pictures of the endoscopic submucosal resection with a ligation device sequence. **(A)** Marking a neuroendocrine tumor (NET) using the tip of a loop snare with soft-coagulation mode. **(B)** Injecting saline into the submucosa below the NET just before following band ligation. **(C)** The NET ligated with a rubber band involving its surrounding mucosa and submucosa. **(D)** Snaring under the ligation band. **(E)** Cutting the ligated them with forced-coag-1 mode. **(F)** The wound was closed with endoclips as soon as possible after removal of the cap.

The known lesion was identified using a gastroscope (EG-L580RD; Fujifilm) with a black cap (MAJ-1990; Olympus) inserted in the rectum first. The NET was marked with the tip of a snare (SnareMaster SD-221U-25; Olympus) using 30 watt soft-coagulation mode if viewing the NET would be unclear after injection (Figure 3A). Then the gastroscope was withdrawn. The cap of a ligation device (Pneumo-activate EVL device; Sumitomo Bakelite Corp., Tokyo, Japan) was put at the tip of the gastroscope instead of the black cap and a ligation band set on the cap. The endoscope was inserted up to the NET again. Saline was injected into the submucosa below the NET to decrease the coagulation effect on the muscularis and to facilitate ligation using an injection needle (M-Jector needle IN-25M; Medicos Hirata Inc., Tokyo, Japan) (Figure 3B). We aspirated the NET with its surrounding mucosa and submucosa into the cap as much as possible and subsequently deployed the ligation band (Figure 3C). The ligated specimen was snared, placing the snare (SnareMaster SD-221U-25; Olympus) under the ligation band as soon as possible and cut with 15 watt forced-coag-1 mode (ESG-100; Olympus) (Figure 3D). The mucosal defect created by resection was closed with endoclips (16 mm SureClip and/or Micro-Tech and EZ clip; Olympus) immediately (Figures 3E,F).

### Evaluation of Endoscopic Resection

All procedures were recorded on video. Procedure time of UEMR was measured from the start of water immersion after endoscopic ultrasound to completion of closure of the mucosal defect on video. Procedure time of ESMR-L was recorded from injection to completion of closure on video. The total cost of devices used in each procedure was calculated referring to a standard price list for devices (Table 1).

**TABLE 1:** Comparison of device cost for underwater endoscopic mucosal resection without submucosal injection (UEMR) and endoscopic submucosal resection with a ligation device (ESMR-L).

	UEMR	Price	ESMR-L	Price
Attachment	Disposal distal attachment	$18.20	Pneumo-activate EVL device Black cap	$137.30 $18.20
Snare	RotaSnare	$40.90	SnareMaster	$50.00
Injection	None		M-jector needle	$40.90
Clip	SureClip	$31.80	SureClip	$31.80
	EZ Clip	$8.90	EZ Clip	$8.90

*Prices shown are in US$, US$1.00 = 110 Japanese Yen.*

The size, location, resection time, and pathological findings for each lesion were evaluated after resection. *En bloc* resection was defined as a tumor removed as a single piece. R0 resection was defined as an *en bloc* resection with pathologically negative resection margins. The definition of perforation included perforations both during and after the procedure. Postoperative bleeding was defined as hematochezia with a decrease in hemoglobin concentration > 2 g/dL, requiring transfusion or requiring endoscopic hemostasis within 14 days of the procedure.

### Statistical Analysis

The Mann-Whitney *U*-test was used to assess non-parametric data. The chi-square test was used to evaluate categorical data. If less than 5 expected values were in a group when evaluating the categorical data, the Fisher’s exact test was used. Statflex version 7.0 software (Artech Co., Ltd., Osaka, Japan) was used. Differences were considered significant when *P* < 0.05.

## Results

### Baseline Characteristics of Thirty Two Patients

Thirty-two patients were divided into the UEMR group (*n* = 7) and the ESMR-L group (*n* = 25). Age and gender were similar between the two groups (Table 2). All endoscopic procedures were performed under the direction of endoscopists who are board certified by The Japan Gastroenterological Endoscopy Society. UEMR procedures were performed on an outpatient basis for all patients, and 92% of ESMR-L (23/25) procedures were performed as inpatient procedures [median (IQR), days 4 (3–4)]. Biopsies were performed in three patients (43%) in the UEMR group and in 17 (68%) of the ESMR-L group (*p* = 0.379) before resection. All biopsies revealed a histopathological diagnosis of NET.

**TABLE 2:** Comparison of characteristics and outcomes of underwater endoscopic mucosal resection without submucosal injection (UEMR) and endoscopic submucosal resection with a ligation device (ESMR-L).

	UEMR (*n* = 7)	ESMR-L (*n* = 25)	*P*-value
Age, years, median (IQR)	65 (50.5–67.5)	63 (49–68)	0.837
Gender (male/female), n	4/3	17/8	0.667*
Hospitalization, n	0 (0%)	23 (92%)	< 0.001*
Previous biopsy, n	3 (43%)	17 (68%)	0.379*
Size of lesion, mm, median (IQR)	4 (3–5)	4 (3–5)	1.000
Procedure time, min, median (IQR)	6 (5–8)	12 (9–14)	0.002
*En bloc* resection, n	7 (100%)	25 (100%)	1.000*
R0 resection, n	7 (100%)	25 (100%)	1.000*
Pathological findings, n			
NET (G1) NET (G2)	7 (100%) 0 (0%)	23 (92%) 2 (8%)	1.000*
Adverse events, n			
Immediate perforation Delayed perforation Delayed bleeding	0 (0%) 0 (0%) 0 (0%)	0 (0%) 0 (0%) 2 (8%)	1.000* 1.000* 1.000*
Total cost of devices, median (IQR)	$90.45 (83.64–108.41)	$274.73 (265.86–292.45)	< 0.001

IQR: interquartile range, NET: neuroendocrine tumor. *Fisher’s exact test.

### Therapeutic Outcomes

The procedure time was significantly shorter in the UEMR group than in the ESMR-L group [median (IQR), min, 6 (5–8) vs. 12 (9–14), *p* = 0.002] (Table 2). *En bloc* resection and R0 resection rates were 100% in both groups. Pathological evaluations were predominantly NET G1 in both groups. Two patients in the ESMR-L group developed delayed bleeding controlled by endoscopic hemostasis. The device cost was significantly higher in the ESMR-L group than the UEMR group by approximately US$180 [median (IQR), $90.45 (83.64–108.41) vs. $274.73 (265.86–292.45), *P* < 0.001].

## Discussion

Although ESMR-L and ESD have been commonly used for endoscopic resection of rectal NETs in Japan, underwater EMR was only recently described (6). The present study compares UEMR with ESMR-L and demonstrates similar *en bloc* resection, R0 resection, perforation and delayed bleeding rates for the two groups. The procedure time for UEMR was significantly shorter than ESMR-L, and the cost of equipment for UEMR was significantly less. UEMR does not require complicated devices or techniques and uses only water immersion. Therefore, UEMR may be performed much more efficiently than ESMR-L. These data support the use of a relatively simple endoscopic resection technique to remove rectal NETs that are increasingly being detected and avoid more complicated and expensive endoscopic resection techniques (e.g., ESD and ESMR-L) for these lesions.

In water immersion without submucosal injection, the submucosal layer is thickened which facilitates resection with a thick submucosal layer even without ligation devices (6, 7). When endoscopically resecting rectal NETs smaller than 5 mm in Japan, the cost is covered by the national health insurance system the same as ordinary EMR or ordinary polypectomy. The equipment cost for UEMR is significantly lower than for ESMR-L. UEMR is the most reasonable technique for endoscopic resection, especially for diminutive NETs.

**FIGURE 4:**
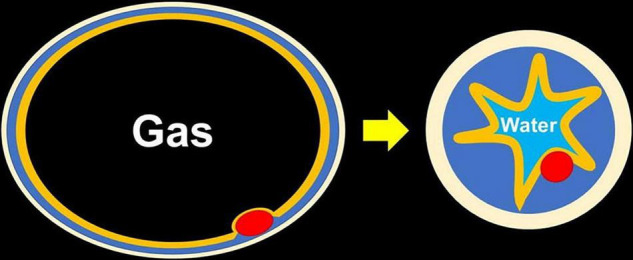
Changing of thickness of the submucosa seen with water immersion. After fully exchanging the gas for water, the muscularis becomes circular and contracted, which makes the submucosa thickened and easy to be snared even without submucosal injection.

**FIGURE 5:**
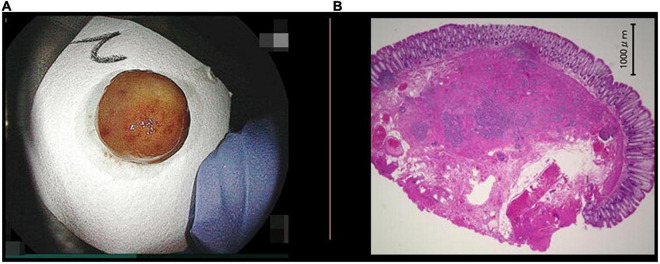
Pathology of a resected neuroendocrine tumor (NET). **(A)** The specimen of a NET resected with underwater endoscopic mucosal resection, 1 cm in diameter. A NET is identified as a 3 mm yellow submucosal tumor with an obviously negative horizontal margin. The specimen was put on a piece of filter paper with its stump down to be fixed to avoid stump shrinkage. **(B)** Specimen with hematoxylin and eosin stain showed thick submucosa under the NET and a negative vertical margin. There was no lymph vessel invasion but there was slight venous invasion. The rossete-forming cells observed at low-magnification (hematoxylin and eosin stain, 20×).

The most important technical point in resecting rectal NETs is to obtain a negative vertical margin because rectal NETs originate from the deep portion of the lamina propria and expansively grow toward the submucosa through the muscularis mucosa. Therefore, ESD or ESMR-L have been used to excise the deep submucosal layer for resecting rectal NETs, and the superiority of ESMR-L compared to ESD was reported with regard to R0 resection rate and procedure time (9). When performing UEMR for large sessile colorectal polyps, a thickened submucosal layer surrounded by a contracted round muscularis facilitates obtaining an adequate vertical margin (Figure 4) (6). A recent case series reported that UEMR yields a high R0 resection rate when resecting rectal NETs (7). Although all patients in this study achieved an R0 resection, considering further options in case of resection with an obscure pathological margin after UEMR or ESMR-L is important. When evaluating the vertical resection margin of a NET after ESMR-L or UEMR, fixation of the resected specimen needs to be done with particular care to avoid mechanical damage. Pinning of the resected specimen with excessive extension can thin and destroy the resected submucosa between the NET and the cut end, resulting in a falsely positive vertical margin. Therefore, excessive tension should be avoided when fixing the NET specimen (Figures 5A–C).

A disadvantage of UEMR is a narrowed visual field caused by a change in the refractive index of light and a narrowed intestinal lumen between the shrunken mucosa with water immersion. The narrow visual field makes securing the lateral margin difficult. However, when the rectal NET is small, it can be snared while confirming an adequate lateral margin even with a narrow visual field. Placing a transparent cap at the tip of the endoscope keeps the visual field from the shrunken mucosa. Even if we cannot be confident to have snared the entire rectal NET, we can snare it repeatedly until it is definitely captured in the snare loop, just like performing PP-CUE (progressive polyp contraction with underwater endoscopic resection) which we reported previously (10).

A study comparing UEMR and ESD for resecting rectal NETs showed similar rates for R0 resection and adverse events, and demonstrated a significantly shorter procedure time for UEMR compared to ESD (8). Similar to resecting superficial colorectal flat tumors, rectal NETs do not have increased vascular supply or a muscle retraction that increases the risk of perforation. We believe that the safety of resecting rectal NETs using UEMR is similar to that for superficial colorectal flat tumors.

We used “pure cut” mode without any coagulation to diminish the risk of delayed bleeding during UEMR. Coagulation mode has the potential to extend a post-coagulation ulcer after resection resulting in delayed bleeding or perforation. Although cut mode may induce intraprocedural bleeding, bleeding during the procedure is easily controlled compared to delayed bleeding. Therefore, pure cut mode is used in outpatient endoscopic interventions.

We recognize that there are acknowledged limitations. First, this is a retrospective study from one tertiary care center. Second, there is a learning curve in establishing the UEMR procedure and handling of resected specimens in both groups for appropriate pathological evaluation. Third, evaluation of the risk of lymph node metastases from rectal NETs is not as fully established as for superficial rectal cancers. Fourth, this study had a small sample size (32 patients) including seven patients in the UEMR group. This may affect the power of the study. Despite this limitation, there is a significant difference in the procedure time and cost of the procedures between the two techniques.

## Conclusion

UEMR demonstrates similar resection results with a shorter procedure time and lower cost compared to ESMR-L. We report the first comparison of UEMR with ESMR-L. UEMR can become the first choice to resect rectal NETs < 10 mm. Further study is necessary to confirm this preliminary result.

## Data Availability Statement

The raw data supporting the conclusions of this article will be made available by the authors, without undue reservation.

## Ethics Statement

The studies involving human participants were reviewed and approved by the Institutional Review Board of Jichi Medical University (No. 20-103). Written informed consent for participation was not required for this study in accordance with the national legislation and the institutional requirements.

## Author Contributions

All authors listed have made a substantial, direct, and intellectual contribution to the work, and approved it for publication.

## Conflict of Interest

The authors declare that the research was conducted in the absence of any commercial or financial relationships that could be construed as a potential conflict of interest.

## Publisher’s Note

All claims expressed in this article are solely those of the authors and do not necessarily represent those of their affiliated organizations, or those of the publisher, the editors and the reviewers. Any product that may be evaluated in this article, or claim that may be made by its manufacturer, is not guaranteed or endorsed by the publisher.

## Supplementary Material

Supplementary Video 1 Procedure of underwater endoscopic mucosal resection (UEMR) for a rectal neuroendocrine tumor (NET).
